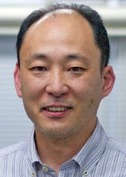# Carbometalation and Heterometalation of Carbon‐Carbon Multiple‐Bonds Using Group‐13 Heavy Metals: Carbogallation, Carboindation, Heterogallation, and Heteroindation

**DOI:** 10.1002/asia.201901730

**Published:** 2020-02-18

**Authors:** Yoshihiro Nishimoto, Makoto Yasuda

**Affiliations:** ^1^ Department of Applied Chemistry Graduate School of Engineering Osaka University 2-1 Yamadaoka, Suita 565-0871 Osaka Japan

**Keywords:** Carbometalation, Gallium, Heterometalation, Indium, Metalation

## Abstract

Organogallium and ‐indium compounds are useful reagents in organic synthesis because of their moderate stability, efficient reactivity and high chemoselectivity. Carbogallation and ‐indation of a carbon‐carbon multiple bond achieves the simultaneous formation of carbon‐carbon and carbon‐metal bonds. Heterogallation and ‐indation construct carbon‐heteroatom and carbon‐metal bonds. Therefore, these reaction systems represent a significant synthetic method for organogalliums and ‐indiums. Many chemists have attempted to apply various types of unsaturated compounds such as alkynes, alkenes, and allenes to these reaction systems. This minireview provides an overview of carboindation and ‐gallation as well as heteroindation and ‐gallation.

## Graphical abstract

**Organogallium and ‐indium compounds** are useful reagents in organic synthesis due their moderate stability. Carbogallation and ‐indation of carbon‐carbon multiple‐bonds achieves simultaneous formation of carbon‐carbon and carbon‐metal bonds. And, heterogallation and ‐indation are used to construct carbon‐heteroatom and carbon‐metal bonds. So, these reaction systems represent significant synthetic methods for the use of organogalliums and ‐indiums. Various unsaturated compounds, such as alkynes, alkenes, and allenes are applicable to these reaction systems to access to organogalliums and ‐indiums with diverse functionalities.

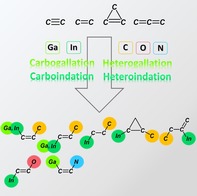

## 1 Introduction

Carbometalation of a carbon‐carbon multiple‐bond is an important and powerful method for the synthesis of organometallic compounds because organometallics are produced by the formation of a new carbon‐carbon bond.^1^ There are many types of transition metal‐catalyzed carbometalations, and most of them occur in a *syn*‐addition fashion. Transition metal catalyst‐free carbometalation is also an attractive reaction because toxic and expensive transition metals are not required. In several reports, highly reactive organometallic compounds such as organolithiums and Grignard reagents have been added directly to alkynes and alkenes. However, the high nucleophilicity of the organometallics that were used led to a lack of functional group tolerance. On the other hand, carbometalation using group‐13 heavy metal species such as organogalliums and ‐indiums is a diverse reaction system with high chemoselectivity. This is because the Ga(III) and In(III) centers possess moderate Lewis acidity and high π‐electron affinity that is caused by the large ionic radius, which leads to a compatibility with functional groups and to the activation of carbon‐carbon multiple bonds, respectively.^2^ Moderate reactivity of organogalliums and ‐indiums enables chemoselective reactions, and the organometallics produced by carbometalation are applicable to sequential reactions.^1^, ^3^ Carboindation via a radical mechanism is possible due to the stability of low‐valent indium species. Therefore, the importance of carbogallation and carboindation has increased because of their usability and diversity. This review focuses on stoichiometric carbogallation and carboindation to synthesize organogalliums and organoindiums, respectively, and the application of these organometallic compounds to organic synthesis. Many excellent catalytic reactions, in which the catalytic cycle involves carbogallation and ‐indation, have been reported. In these cases, organogalliums and indium species are generated as transient intermediates, but are not afforded as final products. Therefore, the catalytic reactions are excluded in this review.^2^ Additionally, heterometalation of carbon‐carbon multiple bonds (heterogallation and heteroindation) is described. This is the reaction wherein new carbon‐hetero atom bonds and new carbon‐metal bonds are formed via the addition of hetero and metal atoms to the multiple bond.

## 2 Carbogallation of Carbon‐Carbon Multiple‐Bonds

### 2.1 Carbogallation with Organogalliums

**Scheme 1:**
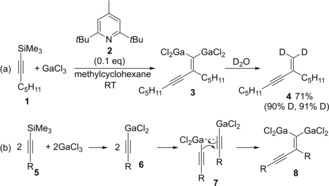
Carbogallation between alkynylgalliums generated by transmetalation of alkynylsilanes with GaCl_3_.

The first carbogallation of alkynes was reported by Yamaguchi.^4^ Treatment of alkynyltrimethylsilane with GaCl_3_ in the presence of a catalytic amount of pyridine **2** gave dimeric product **4** after a workup with D_2_O, and two deuterium atoms were introduced at an *exo*‐methylene moiety of **4**, which suggested the possibility of a generation of **3** via carbogallation (Scheme 1a). The addition of pyridine **2** prevented origomerization of **4**. The reaction mechanism is shown in Scheme 1b. Transmetalation between **5** and GaCl_3_ produces alkynylgallium **6**, and then carbogallation between two alkynylgallium **6** yields digallium compound **8**.

**Scheme 2:**
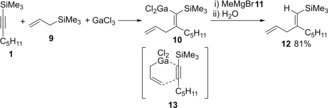
Allylgallation of alkynylsilane with allylic gallium generated by transmetalation between allylic silane and GaCl_3_.

**Scheme 3:**
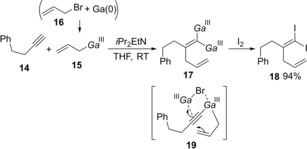
Allylgallation of terminal alkynes with allylic gallium generated from allylbromide and Ga(0).

Allylgallium species generated by transmetalation between allylsilane **9** and GaCl_3_ underwent *syn*‐carbogallation (**13**) of alkynylsilane **1** (Scheme 2).^5^ Takai reported that an allylgallium generated from allyl bromide **16** and Ga(0) was applicable to carbogallation of terminal alkynes (Scheme 3).^6^ After alkyne **14** was reacted with allylic gallium **15**, quenching with I_2_ gave 1,1‐diiodoalkene **18**. Authors proposed the Ga(III)‐assisted carbogallation of allyl alkynylgallium species **19**.

**Scheme 4:**
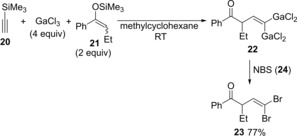
Allylgallation of alkynylsilane with allylic gallium generated by transmetalation between allylic silane and GaCl_3_.

Yamaguchi reported that carbogallations of silyl acetylene **20** proceeded using GaCl_3_ and silyl enolates (Scheme 4). Carbogallation of silylacetylene **17** with GaCl_3_ and silyl enol ether was discovered.^7^, ^8^, ^9^ Quenching with NBS (**24**) gave 1,1‐dibromoalkene **23** (Scheme 4), which indicated the production of 1,1‐dimetalated alkene **19** by carbogallation.

**Scheme 5:**
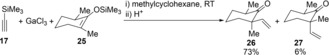
Carbogallation using silyl enol ether **25** derived from a six‐membered cyclic ketone.

**Scheme 6:**
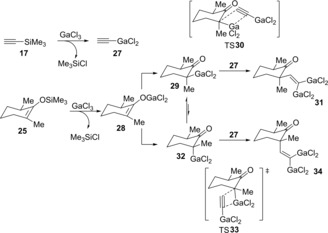
Plausible reaction mechanism for carbogallation using silyl enolates and GaCl_3_.

Carbogallation using silyl enol ether **25**, which is derived from a six‐membered cyclic ketone, predominantly provided ethenylated cyclic ketone **26** with a equatorial vinyl group (Scheme 5).^10^ Enolate ethenylation and alkylation display equatorial stereochemistry and axial stereochemistry, respectively. It is proposed that α‐gallioketone is the reactive species rather than gallium enolate (Scheme 6). **25** and silyl acetylene **17** transmetalate with GaCl_3_ to provide gallium enolate **28** and gallium acetylide **27**, respectively. **28** isomerizes to α‐gallioketones **29** and **32**. There is an equilibrium between **29** and **32**, and **29** has a bulky GaCl_2_ group at the equatorial position, which makes it more stable than **32**. Then, carbogallation of **27** with **29** preferentially proceeds to give 1,1‐digallioalkene **31**.

**Scheme 7:**
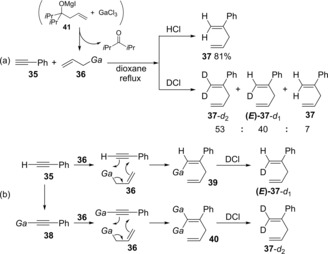
Carbogallation of alkynes using allylic galliums produced by retro‐allylation of homoallylic alcohols with GaCl_2_.

Yorimitsu and Oshima disclosed carbogallation of alkynes using allylic galliums generated by retro‐allylation (Scheme 7a).^11^ Allylic gallium **36** was produced by retro‐allylation between homoallyl alkoxide **41** and GaCl_3_, and then reacted with alkyne **35** to give product **37** after quenching with an aqueous solution of HCl. Quenching with DCl instead of HCl afforded di‐ and monodeuterated products (**37**‐*d*_2_ and (*E*)‐**37**‐*d*_1_). Based on a DCl‐quenching experiment, a *syn*‐addition mechanism was proposed (Scheme 7b). Allylgallation of alkyne **35** with **36** proceeds via a six‐membered transition state to give alkenylgallium **39**. Meanwhile, deprotonation of alkyne **35** by basic allylic gallium gives alkynylgallium **38**. The *syn*‐addition of **36** to **38** yields 1,1‐metalated alkene **40**.

**Scheme 8:**
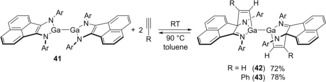
Synthesis of (dpp‐bian)Ga−Ga(dpp‐bian) complex by carbogallation.

1,2‐Bis(arylimino)acenaphthene (bian) ligands have attracted much attention. The synthesis of (dpp‐bian)Ga−Ga(dpp‐bian) complex **41** and reversible carbogallation of alkynes with **41** was reported (Scheme 8).^12^ When treatment of a solution of **41** with acetylene or phenylacetylene was carried out, the Ga−N−C fragment was added to the alkynes to provide carbon‐carbon and carbon‐gallium bonds and to give alkenyl gallium **42** or **43**, respectively. These organogalliums were identified by single‐crystal X‐ray analysis. The carbogallation was reversible, and the equilibrium between **43** and **41**+phenyl acetylene was studied by ^1^H NMR spectroscopy.

**Scheme 9:**
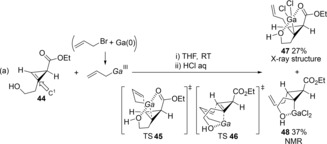
Carbogallation of cyclopropenes using allylic galliums.

Carbogallation of a carbon‐carbon double bond was established using allylgallium species. Araki reported a regioselective allylgallation of cyclopropenes (Scheme 9).^13^ The reaction of the allylic gallium with cyclopropene **44** bearing a hydroxyalkyl group on the C^1^ carbon gave cyclopropylgallium products **47** and **48**. The structure of **47** was revealed by X‐ray diffraction analysis. Therefore, the coordination of the hydroxy group to a Ga atom in the allylic gallium was classified as *anti*‐Markovnikov regioselectivity (TS **45** and TS **46**).

### 2.2 Carbogallation of Gallium Trihalide‐Activated Carbon‐Carbon Multiple‐Bond

**Scheme 10:**
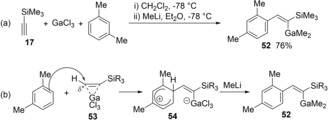
Carbogallation of silyl acetylene with GaCl_3_ and nucleophilic arenes.

**Scheme 11:**
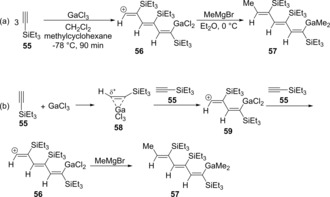
Trimerization of silyl acetylene via carbogallation.

A reaction of silyl acetylene with GaCl_3_ and nucleophilic arenes was carried out, followed by treatment with MeLi to gave alkenyldimethylgallium **52** (Scheme 10a).^14^ π‐Complex **53** was formed from GaCl_3_ and vinyl *tert*‐butyldimethylsilane and identified at −78 °C via NMR spectroscopy (Scheme 10b). Carbogallation proceeds via the regioselective nucleophilic attack of an arene at the β‐carbon atom of a silyl group to give zwitterion intermediate **54**. Finally, a proton abstract and ligand exchange by MeLi produce alkenylgallium **52**. In the absence of nucleophilic arenes, ethynylsilane **55** was trimerized via alkenylgallation caused by GaCl_3_ (Scheme 11a).^15^ The reaction of GaCl_3_ with 3 equivalents of **55** in CH_2_Cl_2_ and methylcyclohexane at −78 °C gave trienyl cation **56**. Interestingly, the cation intermediate **56** was identified by ^1^H and ^13^C NMR spectroscopies. MeMgBr in Et_2_O was then added to the solution of **56** to produce alkenylgallium **57**. Proposed mechanism is shown in Scheme 11b. The reaction is initiated with the activation of **55** by GaCl_3_, and then the nucleophilic addition of another **55** gives alkenyl cation **59**. The cation **59** is converted to trienyl cation **56** by the addition of **55**. Finally, the treatment of MeMgBr produces trienylgallium compound **57**.

**Scheme 12:**
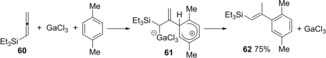
Carbogallation of silyl allene with GaCl_3_ with *p*‐xylene.

Silyl allene **60** also underwent carbogallation with GaCl_3_ and *p*‐xylene (Scheme 12).^16^ In this case, however, an intramolecular proton transfer in zwitterion alkylgallium species **61**, which was formed by the carbogallation, occurred to give alkenylsilane **62** and GaCl_3_, so a stable organogallium product was not obtained.

**Scheme 13:**
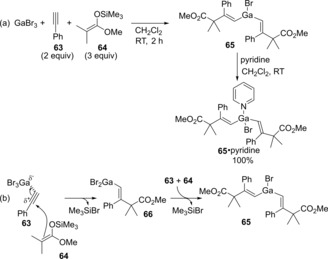
Regio‐ and stereoselective *anti*‐carbogallation of alkynes using GaBr_3_ and silyl ketene acetals.

We reported the regio‐ and stereoselective *anti*‐carbogallation of alkynes using GaBr_3_ and silyl ketene acetals (Scheme 13).^17^ Alkyne **63** was treated with GaBr_3_ and silyl ketene acetal **64** to give dialkenylgallium **65** (Scheme 13a). The structure of **65** was determined by X‐ray diffraction analysis after complexation with pyridine (**65**⋅pyridine). That result suggested carbogallation occurred as shown in Scheme 13b. The interaction between GaBr_3_ and a carbon‐carbon triple bond of alkyne **63** causes the regioselective nucleophilic attack of **64** from the opposite site of GaBr_3_ to provide monoalkenylgallium **64** and Me_3_SiBr.

**Scheme 14:**
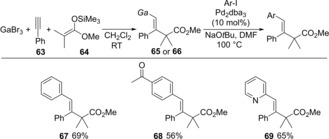
Regio‐ and stereoselective synthesis of trisubstituted alkenes by using carbogallation/cross‐coupling sequential process.

Synthesized alkenylgalliums were directly applied to Pd‐catalyzed cross‐coupling with aryl iodides (Scheme 14). Various types of functional groups were compatible with alkenylgalliums, and 4‐acetyliodobenzene, 2‐iodopyridine as well as iodobenzene smoothly coupled with alkenylgalliums (**65** or **66**) to give the corresponding trisubstituted alkene products (**67**, **68**, and **69**). The use of phosphine ligands for a Pd‐catalyst is not necessary to the cross‐coupling of organogalliums (and organoindiums) in highly‐coordinative solvents such as DMF perhaps because the solvents could work as efficient ligands.

**Scheme 15:**
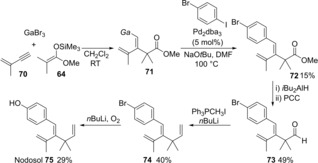
The first total synthesis of nodosol via carbogallation/cross‐coupling sequential process.

The developed process for trisubstituted alkene synthesis via carbogallation/cross‐coupling was employed for the first total synthesis of nodosol **75** (Scheme 15). The key synthetic intermediate, diene **72**, was regio‐ and stereoselectively prepared by carbogallation of enyne **70** followed by cross‐coupling using 4‐bromoiodobenzene.

**Scheme 16:**
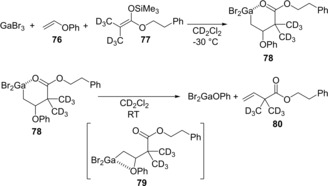
Carbogallation of vinyl ether **76** with GaBr_3_ and silyl ketene acetal **77**, and β‐elimination of phenoxygallium to give α‐vinyl ester **80**.

We discovered that vinyl ether **76** underwent carbogallation with GaBr_3_ and silyl ketene acetal **77** at low temperature to give *β*‐phenoxyalkylgallium species **78** (Scheme 16).^18^ Interestingly, the *syn*‐elimination of phenoxygallium from **78** via transition state **79** occurred at room temperature to give α‐vinyl ester **80**.

**Scheme 17:**
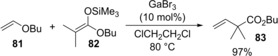
Catalytic coupling reaction of vinyl ether **81** with silyl ketene acetal **82**.

**Figure 1:**
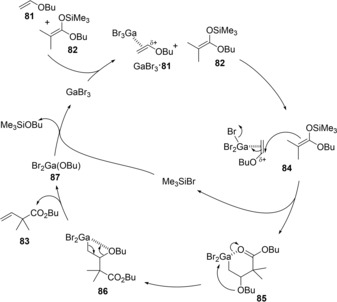
Proposed mechanism of GaBr_3_‐catalyzed cross‐coupling between vinyl ethers with silyl ketene acetals.

Therefore, the authors established the first catalytic cross‐coupling of alkenyl ethers with silyl ketene acetals. Vinyl ether **81** was coupled with silyl ketene acetal **82** in the presence of GaBr_3_ catalyst to produce α‐vinyl ester **83** (Scheme 17). Silyl ketene imines were also applicable to this cross‐coupling system.^19^ A proposed catalytic cycle is shown in Figure 1. GaBr_3_ is coordinated by alkenyl ether **1**, and then the anti‐carbogallation of the activated **81** with silyl ketene acetal **82** occurs regioselectively to give alkylgallium **85** and Me_3_SiBr. After conformation change from six‐membered **85** to four‐membered **86**, *syn*‐elimination of Br_2_GaOBu proceeds to give coupling product **83**. Finally, GaBr_3_ is regenerated by transmetalation between Br_2_GaOBu and Me_3_SiBr.

## 3 Heterogallation of Carbon‐Carbon Multiple‐Bonds

**Scheme 18:**
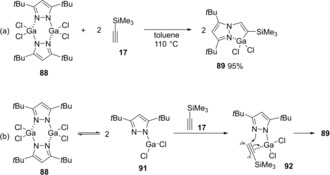
Azagallation of alkyne with pyrazolato gallium dichloride **88**.

Zheng and Yang demonstrated the first synthesis and characterization of pyrazolato gallium dichlorides and its application to azagallation of alkynes.^20^ When pyrazolato gallium dichloride **88** was mixed with silyl acetylene, azagallation of the carbon‐carbon triple‐bond gave pyrazolato alkenylgallium **89** (Scheme 18a). The reaction mechanism remains unclear, but the more reactive three‐coordinated gallium species **91** is proposed (Scheme 18b). The gallium center of **91** activates silyl acetylene by π‐coordination, and the intramolecular nucleophilic attack by a *β*‐nitrogen of the Ga atom causes azagallation.

**Scheme 19:**
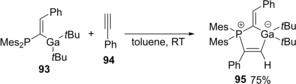
Phosphagallation of a carbon‐carbon triple bond by Ga/P FLPs complex.

Uhl synthesized Ga/P complex **93** with the geminal arrangement of coordinatively unsaturated Ga and P atoms.^21^ When **93** was mixed with alkyne **94**, phosphagallation of a carbon‐carbon triple bond occurred to give five‐membered heterocycle **95** involving P and Ga atoms (Scheme 19). The terminal C atom of alkyne **94** has its relatively high negative partial charge to bind to the electropositive Ga atom, and the relatively positive internal C atom binds to the electronegative P atom.

## 4 Carboindation of Carbon‐Carbon Multiple‐Bonds

### 4.1 Carboindation with Organoindiums

**Scheme 20:**
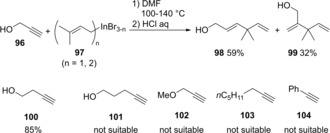
Carboindation of a carbon‐carbon triple‐bond nearby a hydroxy group.

Various carboindations using allylindiums generated by the reaction of In(0) with allylic halides have been reported. Butsugan developed the first carboindation of alkynols in 1992 (Scheme 20).^22^ The carboindation of alkynol **96** with allylic indium **97** proceeded via a syn addition mechanism to give *anti*‐Markovnikov adduct **98** and Markovnikov adduct **99**. The reaction using 3‐butyn‐1‐ol **100** gave a high yield, but 4‐pentyn‐1‐ol **101**, 3‐methoxy‐1‐propyne **102**, 1‐octyne **103**, and phenylacetylene **104** were not suitable to these conditions. Therefore, a hydroxy group near the triple bond is important in the carboindation.

**Scheme 21:**
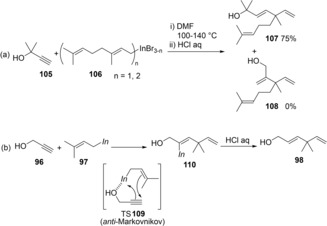
Regioselectivity and plausible mechanism for carboindation of alkynols with allylic indiums.

The regioselectivity depended on the structures of alkynols and allylic indiums (Scheme 21a). The reaction using sterically hindered alkynol **105** and allylic indium **106** showed perfect regioselectivity. A proposed reaction mechanism is shown in Scheme 21b. A hydroxy group coordinates to an indium atom of allylic indium **97**. The allyl group on the coordinated indium atom adds to the terminal carbon of alkynol **96** and the indium adds to the inner carbon.^23^

**Scheme 22:**
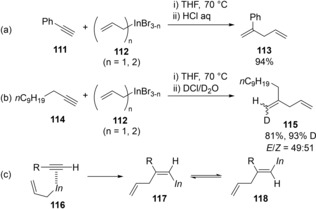
Carboindation of unactivated alkynes using an allylic indium.

Yamamoto^24^ and Ranu^25^ independently reported the carboindation of unactivated alkynes using allylic indiums (Scheme 22). In contrast to the DMF solvent conditions, aromatic alkyne **111** and aliphatic alkyne **114** without a directing group such as a hydroxy group smoothly underwent carboindation using an allylic indium under THF solvent conditions to give dienes **113** and **115**, respectively (Scheme 22a and 22b). Quenching with DCl/D_2_O afforded an *E*/*Z* mixture of deuterated diene product **115** (Scheme 22b). Therefore, the carboindation of an alkyne with an allylic indium proceeds via *syn*‐addition fashion (**116**) to produce alkenyindium **117**, which undergoes *E*‐*Z* isomerization (Scheme 22c).

**Scheme 23:**
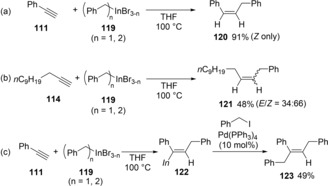
Benzylindation of alkynes using benzylic indium species.

Carboindation of alkynes using benzylic indiums was also reported by Yamamoto (Scheme 23).24b The benzylindation of aromatic alkyne **111** occurred in an *anti*‐addition manner (Scheme 23a), while that of aliphatic alkyne **114** took place in a nonstereoselective fashion (Scheme 23b). As in the case of allylindation, *syn*‐addition followed by *E*‐*Z* isomerization occurred. The produced alkenylindium **122** coupled with benzyl iodide in the presence of a palladium catalyst to give three‐component coupling product **123** in 49 % yield (Scheme 23c).

**Scheme 24:**
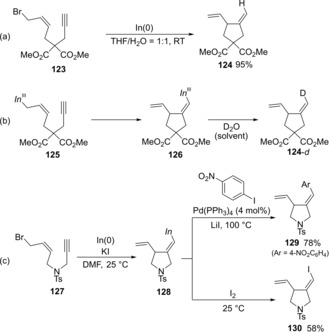
Intramolecular allylindation via the addition of an allylindium moiety to a carbon‐carbon triple bond.

Intramolecular cyclizations of alkynes bearing an allylic bromide moiety via allylindation were reported. Salter discovered that In(0) mediated the cyclization of **123** to give cyclic compound **124** (Scheme 24a).^26^ The allylic indium moiety of **125**, which generated by the reaction of allylic bromide **123** with In(0), adds to an intramolecular carbon‐carbon triple bond in a *syn* fashion, giving alkenylindium **126**, regio‐ and stereoselectively (Scheme 24b). Actually, the use of D_2_O instead of H_2_O stereoselectively gave deuterated product **124**‐*d*. Lee reported an improved intramolecular cyclization system (Scheme 24c).^27^ The cyclization of **127** in DMF smoothly proceeded without an H_2_O co‐solvent, and the addition of KI was a key factor. The produced alkenylindium 128 was successfully coupled with an aryl iodide or I_2_.

**Scheme 25:**
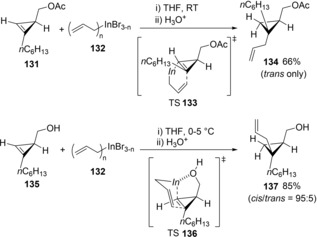
Allylindation of cyclopropenes by allylic indiums and its stereoselectivity controlled by a functional group.

Araki and Butsugan discovered the stereodivergent allylindation of cyclopropene derivatives (Scheme 25).^28^ In a reaction of cyclopropene **131** with allylic indium **132**, the allylic indium was added preferentially from the anti‐face of the acetoxymethyl group (TS **133**) to avoid steric repulsion with the acetoxymethyl group, and the allylic group was introduced to the substituted carbon of the cyclopropene double bond to give product **134**. In contrast, the stereoselectivity of allylindation into cyclopropene **135** was reversed to that of acetate **131**, although the regioselectivity was not changed. This result suggested that the coordination of the hydroxy group to an allylic indium species led to allylindation from the cis face of the hydroxymethyl group (TS**136**).

**Scheme 26:**
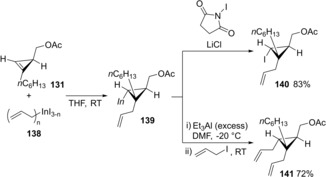
Transformation of cyclopropylindiums produced by carboindation.

The generated cyclopropylindiums were applicable to further transformations (Scheme 26).^29^ The treatment of generated cyclopropylindium **139** by I_2_ and LiCl afforded iodo cyclopropane derivative **140**. In addition, cyclopropylindium **139** coupled with allyl iodide to give diallyl propane **141** in the presence of an excess amount of Et_3_Al, in which a kind of cyclopropylindium ate‐complex generated by the reaction of **139** with Et_3_Al would be an active nucleophile.

**Scheme 27:**
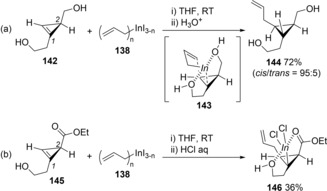
Inverse of regioselectivity by coordination of a hydroxy group in carboindation of cyclopropenes.

Interestingly, the allylindation of cyclopropene **142** bearing a hydroxyalkyl group at a 1‐position as well as at a 2‐position took place with the opposite regioselectivity in the reaction of **135** (Scheme 27a).^30^ The coordination of a hydroxy group hanging on the 2‐position to an indium center of **142** in TS**143** caused a drastic change in the regioselectivity. The X‐ray crystal structure of cyclopropylindium **146** synthesized by allylindation of cyclopropene **145** bearing 2‐hydroxyethyl and ester groups at the C^1^ and C^2^ carbons, respectively (Scheme 26b).

**Scheme 28:**
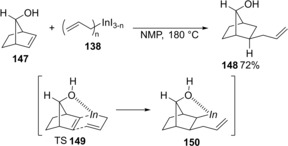
Carboindation of norbornenol **147** with allylic indium **138**.

Other strained olefins underwent carboindation with allylic indium reagents. Allylindation of norbornenol **147** regio‐ and stereoselectively proceeded to give allylated product **148**, and the allylic group was installed exclusively from the *exo* face (Scheme 28).^31^ Therefore, the hydroxy group of **147** acts as a director to lead the carboallylation on the *exo* face (TS **149**).

**Scheme 29:**
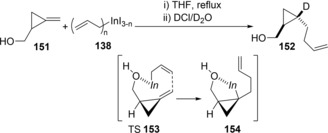
Carboindation of methylenecyclopropane **10** with allylic indium **2**.

The reaction of methylenecyclopropane **151** with allylic indium species **138** exclusively gave deuterated cyclopropane **152** after carrying out a 1 M DCl/D_2_O quench (Scheme 29).^32^ Regio‐ and setereoselective allylindation occurred owing to the coordination of a hydroxyl of **151** to an indium center (TS**153**).

**Scheme 30:**
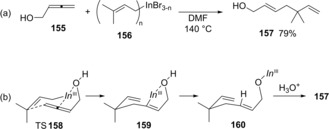
Carboindation of allenols using an allylic indium species.

Araki and Butsugan developed carboindation of allenols using an allylic indium species.^33^, ^34^ The regio‐ and stereoselective addition of prenylindium species **156** to an allene moiety of allenol **155** proceeded to afford product **157** (Scheme 30a). *O*‐protected allenols were not applicable to this carboindation system, which suggested the importance of an hydroxy group for effective carboallylation. A plausible reaction mechanism is shown in Scheme 30b. The carboindation regio‐ and stereoselectively proceeds through hydroxyl‐chelated bicyclic transition state TS**158** to give alkenylindium **159**, and **159** was protonated by an internal hydroxy group to afford indium alkoxide **160**.

### 4.2 Carboindation of Indium Trihalide‐Activated Carbon‐Carbon Multiple‐Bond

**Scheme 31:**
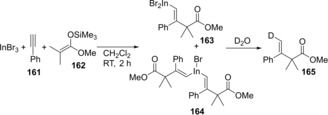
Carboindation of alkynes using InBr_3_ and silyl ketene acetals.

We reported the carboindation of alkynes using InBr_3_ and silyl ketene acetals.^35^ When alkyne **161** was treated with InBr_3_ and silyl ketene acetal **162**, carboindation regio‐ and stereoselectively occurred to give alkenylindiums **163** and **164** (Scheme 30). The treatment of **163** and **164** with D_2_O afforded deuterated **165**. The reaction mechanism is illustrated in Scheme 31. The activation of alkyne **161** by InBr_3_ increase the positive charge on the internal carbon of **161**. The nucleophilic attack by silyl ketene acetal **162** to the internal carbon from the opposite side of the coordinated InBr_3_ to give monoalkenylindium **163**. The successive addition of the resulting **163** to another **161** afforded dialkeynylindium **164** with the same selectivity. The moderate Lewis acidity and high π‐electron affinity of InBr_3_ plays an important role in the effective activation of alkynes in the presence of coordinative ketene silyl acetals. In contrast, the use of strong Lewis acids such as AlCl_3_ and BF_3_⋅OEt_2_ strongly interacted with an oxygen atom of ketene silyl acetals, which resulted in no reaction.

**Scheme 32:**
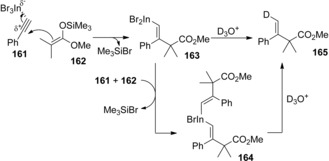
Plausible reaction mechanism for carboindation of alkyne **161** using InBr_3_ and silyl ketene acetal **162**.

Scheme 32 illustrates the *anti*‐carboindation mechanism. The activation of alkyne **161** by InBr_3_ takes place to increase the positive charge on the internal carbon of the alkyne. Ketene silyl acetal **162** attacks the internal carbon from the opposite side of the InBr_3_ to give monoalkenylindium **163**. The successive addition of **163** to another alkyne **161** produces dialkeynylindium **164**. Quenching with D_2_O affords deuterated compound **165**.

**Scheme 33:**
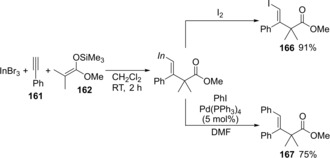
Transformation of alkenylindiums synthesized by carboindation.

The treatment with I_2_ gave iodinated β,γ‐unsaturated ester **166**, and Pd‐catalyzed cross‐coupling of the synthesized alkenylindium with iodobenzene in a one‐pot manner gave coupling product **167** (Scheme 33). In both reactions, the configuration of the corresponding alkenylindium was retained.

**Scheme 34:**
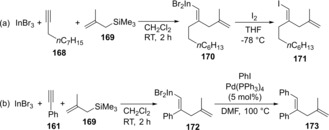
*Anti*‐allylindation of alkynes using InBr_3_ and allylic silanes.

The regio‐ and stereoselective carboindation of alkynes using InBr_3_ and allylic silanes was developed.^36^ This is the first report of the stereoselective *anti*‐allylindation of alkynes. The carboindation of 1‐decyne **168** followed by the quenching of alkenylindium **170** with I_2_ gave **171**, regio‐ and stereoselectively (Scheme 34a). The produced 1,4‐dienylindium **172** was applicable to Pd‐catalyzed cross‐coupling (Scheme 34b).

**Scheme 35:**
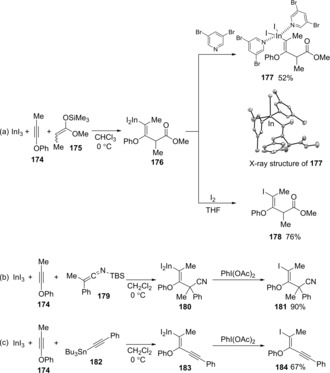
*Anti*‐carboindation of alkynyl ethers using InBr_3_ and organosilicon‐ or stannane compounds.

We also established *anti*‐carboindation of alkynyl ethers using InI_3_ and organosilicons or –stannanes (Scheme 35).^37^ The interaction between InI_3_ and an alkynyl ether is accelerated by the conjugative electron‐donation of an oxygen atom bonding an alkyne moiety, which revealed by DFT calculations. The carboindation of alkynyl ether **174** using InI_3_ and silyl ketene acetal **175** gave metalated enol ether **176** (Scheme 35a). The iodination of **176** afforded trisubstituted enol ether **178** regio‐ and stereoselectively (Scheme 35b), as well as various nucleophiles such as silyl ketene imine **179**, alkynyl stannane **182** (Scheme 35c and 35d).

**Scheme 36:**
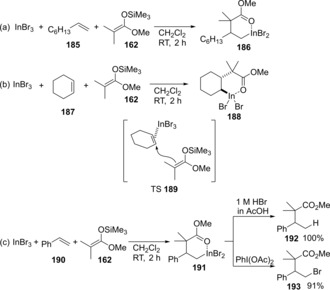
Carboindation of unactivated alkenes and transformation of produced alkylindiums.

An indium trihalide effectively activates simple alkenes (Scheme 36).^38^ The regioselective carboindation of 1‐hexene **185** using InBr_3_ and silyl ketene acetal **162** proceeded to give alkylindium **186** (Scheme 36a). The structure of **186** was revealed by X‐ray diffraction analysis. The crystal structure of alkylindium **188** was afforded by the carboindation of cyclohexene **187** and showed an *anti*‐addition mechanism (TS**189**) (Scheme 36b). Alkylindium **191** was treated with 1 M HBr and PhI(OAc)_2_ to give the corresponding protonated product **192** and brominated product **193**, respectively (Scheme 36c).

### 4.3 Carboindation via Radical Mechanism

**Scheme 37:**
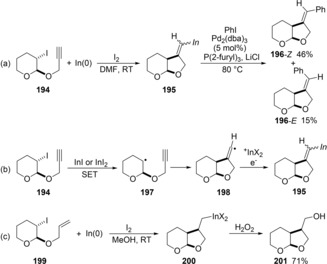
Cyclic carboindation through alkyl radical intermediate produced by reduction of alkyl iodide with row‐valent indium species.

Takemoto discovered indium‐mediated reductive radical cyclization of alkynes bearing an iodoalkane moiety by using a low‐valent indium species (Scheme 35). Treatment of alkyne **194** with In(0) and I_2_ promoted 5‐*exo* cyclic carboindation to give alkenylindium **195** (Scheme 37a).^39^ The generated alkenylindium **195** was coupled with iodobenzene in the presence of a Pd catalyst to give an *E*/*Z* isomer mixture **196**. A proposed mechanism is illustrated in Scheme 37b. The single electron transfer (SET) from a low‐valent indium iodide species, which is generated from In(0) and I_2_, to **194** provides alkyl radical **197**. The radical **197** then undergoes a radical cyclization to produce alkenyl radical **198**, and then the radical reductively combines with an indium cation (^+^InX_2_) to give the *E*/*Z*‐mixture of alkenylindium **195**. Alkene **199** with an iodoalkyl moiety was also applicable to this reductive radical cyclization, and stable alkylindium **200** was isolated (Scheme 37c).^40^ The alkylindium **200** underwent oxidation by H_2_O_2_ to give the corresponding primary alcohol **201**.

**Scheme 38:**
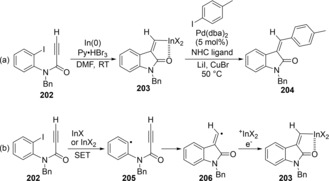
Cyclic carboindation by reduction of aryl iodide with row‐valent indium species.

A reductive radical cyclization of iodoarene bearing an alkynylamide moiety by using In(0)/pyridinium tribromide (PyHBr_3_) occurred regio‐ and stereoselectively to produce 3‐alkylideneoxindoles **203** (Scheme 38a).^41^ In the reaction mechanism (Scheme 38b), either InBr generated from In(0) or InBr_2_ generated from PyHBr_3_ could mediate the radical carboindation of iodoarene **202**, and the coordination of the amide group to an indium atom led to the high stereoselectivity. **202** underwent SET from a low‐valent indium species to afford sp^2^‐σ radical **205**. The radical **205** produces alkenyl radical **206** via radical cyclization, and then the radical exclusively gives an *E*‐isomer of alkenykindium **203** due to the strong coordination of the amido moiety to the indium center. The generated alkenylindium **203** was applied to Pd‐catalyzed cross‐coupling with 4‐iodo toluene.

**Scheme 39:**
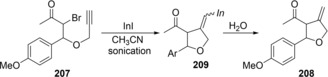
Cyclic carboindation by reduction of α‐bromo carbonyl moiety with row‐valent indium species.

Ranu reported the InI‐mediated cyclization of α‐carbonyl bromo‐alkynes (Scheme 39).^42^ The treatment of α‐carbonyl bromo‐alkyne **207** with InI gave 4‐methylene‐tetrahydrofuran **208** via 5‐*exo* cyclization. Alkenylindium **209** would be produced via InI‐mediated reductive radical carboindation.

**Scheme 40:**
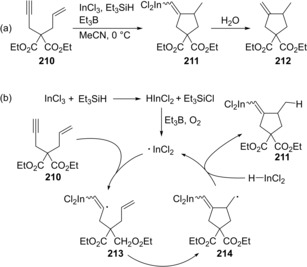
Cyclization of enynes via indium hydride‐mediated radical carboindation.

Shibata and Baba established the carboindation of alkynes and allenes via indium hydride‐mediated radical cyclization. Enyne **210** underwent cyclization in the presence of HInCl_2_, which was generated from InCl_3_ and Et_3_SiH, to give *exo*‐methylene compound **212** through alkenylindium **211** (Scheme 40a).^43^ A proposed mechanism is shown in Scheme 40b. Transmetalation between InCl_3_ and Et_3_SiH gives HInCl_2_, and then the Et_3_B/O_2_ system generates a dichloroindium radical (⋅InCl_2_) from HInCl_2_. The indium radical adds to an alkyne moiety of **210** to produce alkenyl radical **213**. Alkyl radical **214** is produced by the 5‐*exo* cyclization of **213**, and then abstracts a hydrogen atom from HInCl_2_ to give alkenylindium **211**.

**Scheme 41:**
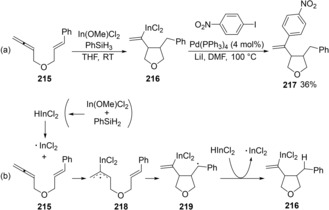
Cyclization of allenynes via indium hydride‐mediated radical carboindation.

Carboindation of allenes by radical cyclization was also developed (Scheme 41a).^44^ When allenene **215** was treated with In(OMe)Cl_2_ and PhSiH_3_, carboindation of an allene moiety and 5‐*exo* cyclization proceeded to give alkenylindium **216**. In this case, an indium radical selectively adds to a central carbon of an allene moiety to provide allylic radical **218** (Scheme 41b). The 5‐*exo* cyclyzation of **218** followed by the hydrogen abstraction of alkyl radical **219** from HInCl_2_ affords alkenylindium **216**. Then, Pd‐catalyzed cross‐coupling of the alkenyl indium **216** with an iodoarene successfully proceeds to yield **217**.

## 5 Heteroindation of Carbon‐Carbon Multiple‐Bonds

**Scheme 42:**
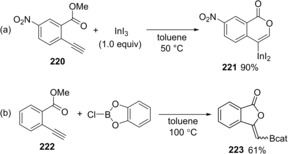
Oxyindation of a terminal alkyne moiety in 2‐alkynyl benzoic ester via 6‐*endo* cyclization.

We reported the regioselective oxyindation of a terminal alkyne moiety in a 2‐alkynyl benzoic ester.^45^ The reaction of 2‐alkynyl benzoic ester **220** with InI_3_ at 50 °C exclusively gave 4‐metalated isocoumarin **221** via oxyindation of an alkyne moiety (Scheme 42a). The 6‐*endo* cyclization contrasts with the 5‐*exo* cyclization caused by B‐chlorocatecholborane (Scheme 42b), which was reported by Blum.^46^ The obtained organoindium **221** was characterized by X‐ray diffraction analysis.

**Scheme 43:**
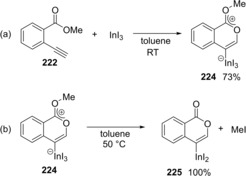
Isolation, characterization, and reactivity of zwitterion intermediate **3**.

**Scheme 44:**
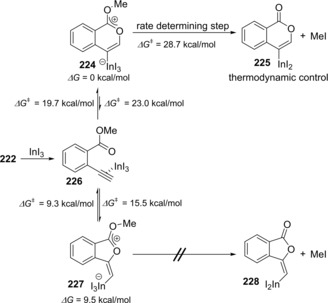
Theoretical calculation study for 6‐*endo* and 5‐*exo* cyclic carboindation.

A reaction mechanism of the oxyindation was revealed by both experimental and theoretical studies. When the reaction of **222** with InI_3_ was carried out at room temperature, zwitterion intermediate **224** with a new carbon‐indium and carbon‐carbon bonds was obtained and identified by X‐ray diffraction analysis (Scheme 43a). Zwitterion **224** was heated at 50 °C, and then elimination of MeI occurred to give isocoumarin **225** bearing a carbon‐indium bond at the 4‐position (Scheme 43b). Based on experimental results, the details of the reaction mechanism were examined using theoretical calculation (Scheme 44), which showed that the activation energy of 5‐*exo* cyclization is much smaller than that of the elimination of MeI so that 5‐*exo* cyclization is reversible. Eventually, selective production of the thermodynamically stable 6‐membered zwitterion **224** produced a remarkable level of 6‐*endo* selectivity.

**Scheme 45:**
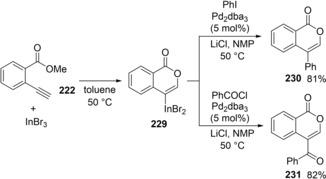
Synthesis of 4‐substituted isocoumarins by oxyindation/cross‐coupling sequential process.

Alkenyl indium **229** was synthesized by the oxyindation of **222** using InBr_3_ and applied to Pd‐catalyzed cross‐coupling with iodobenzene or benzoic chloride in a one‐pot manner to afford 4‐substituted isocoumarin **230** or **231**, respectively (Scheme 45).

**Scheme 46:**
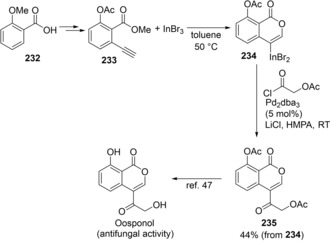
Formal total synthesis of Oosponol.

The formal total synthesis of oosponol was demonstrated by the present oxyindation (Scheme 46). Alkenylindium **234** was synthesized via the oxyindation of **233** with InBr_3_, and then a one‐pot process for the Pd‐catalyzed cross‐coupling of 2‐(acetyloxy)acetyl chloride provided a key isocoumarin precursor, **235**, for Oosponol.^47^

**Scheme 47:**
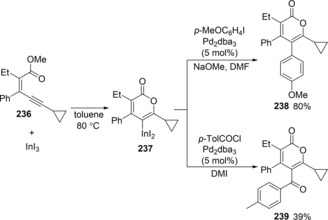
Cyclic oxyindation of carbonyl‐ene‐yne compounds and synthesis of tetrasubstituted pyrones.

**Scheme 48:**
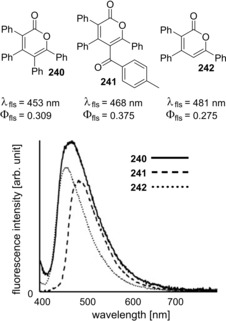
Cyclic oxyindation of carbonyl‐ene‐yne compounds and synthesis of tetrasubstituted pyrones.

Carbonyl‐ene‐yne compounds are also applicable to oxyindation with indium trihalides to give 2‐pyrones bearing a carbon‐indium bond (Scheme 47).^48^ The oxyindation of **236** using InI_3_ produced tetrasubstituted metalated isocumarin **237**. Subsequently, the coupling reaction of **237** with either an aryl iodide or an aroyl chloride in the presence of a palladium catalyst led to 2‐pyrones **238** or **239** bearing four different substituents, respectively. Tetrasubstituted 2‐pyrones **240** and **241** exhibited an aggregation‐induced emission (AIE) in the solid state (Scheme 48). It is noted that **240** and **241** exhibit greater quantum yields than triphenylated 2‐pyrone **242**.^49^

**Scheme 49:**
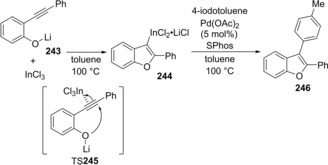
Cyclic oxyindation of lithium *o*‐phenylethynylphenoxide **243** with InCl_3_.

Gomez‐Bengoa and Sestelo reported that cyclic oxyindation of lithium *o*‐phenylethynylphenoxide **243** with InCl_3_ proceeded to give alkenylindium **244** (Scheme 49).^50^ In this case, the π‐coordination of an alkyne moiety to InCl_3_ followed by *endo*‐cyclization induced by the nucleophilic attack of a lithium alkoxide moiety occurs (TS**245**). Organoindium **244** underwent Pd‐catalyzed cross‐coupling with 4‐iodotoluene to afford benzo[*b*]furan **246**. The discovery of oxyindation provided important insight into the reaction mechanism of the In‐catalyzed hydroalkoxylation of *o*‐alkynylphenol derivatives.

## 6 Conclusions and Outlook

We briefly summarized the history of carbogallation and ‐indation, and heterogallation and ‐indation of carbon‐carbon multiple bonds. Carbogallation is divided into two main systems that are the addition of organogallium species and the addition of an external nucleophile to a gallium‐activated alkyne. In the former system, allylgalliums, alkynylgalliums, and gallium enolates were used as organogallium species. In the latter, a gallium trihalide activates a carbon‐carbon multiple‐bond of alkynes, allenes, and alkenyl ethers, and carbogallation is then completed by the nucleophilic addition of various carbon nucleophiles. On the other hand, there are three types of carboindation. Two types are the same as carbogallation. A third type includes a radical pathway, which gives it broader diversity than carbogallation. A third type of carboindation involves a radical mechanism due to the stability of low‐valent indium species. A few types of fascinating azagallation and oxyindation have been established. The moderate reactivity and stability of organogallium and ‐indium has resulted in high levels of compatibility with functional groups. Carbogallation, carboindation, heterogallation, and heteroindation are powerful tools available for the synthesis of highly functinalized organometallic compounds, and further development of this field of study will be extremely useful as more sophisticated organic syntheses are required in the near future.

## Conflict of interest

The authors declare no conflict of interest.

## Biographical Information

*Yoshihiro Nishimoto received his Ph.D. degree in 2009 from Osaka University under the supervision of Prof. Akio Baba. He was appointed Assistant Professor at the Department of Applied Chemistry of Osaka University in 2009. At 2014, he moved to the Frontier Research Base for Global Young Researchers, Center for Open Innovation Research and Education (COiRE). He then became an Associate Professor (2020) at Department of Applied Chemistry of Osaka University. His research interests include the development of Lewis acid‐catalyzed reactions and novel main‐group metal catalysts*.

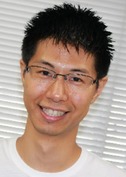

## Biographical Information

*Makoto Yasuda received his Ph.D. degree in 1995 from Osaka University under the guidance of Prof. Akio Baba, and he was appointed Assistant Professor. During 1998–1999, he worked with Prof. J. M. Stryker as a postdoctoral fellow at the University of Alberta. After returning to Osaka University he was promoted to Associate Professor in 2004 and full Professor in 2014. He is currently interested in organic synthesis using main group metals, and in the development of new types of Lewis acids with a designed organic framework. He is also investigating reactive metal species that contribute to stereoselective carbon‐carbon bond formation and their characterization based on spectroscopy and X‐ray crystallographic analysis*.